# Current Perspectives in Set7 Mediated Stem Cell Differentiation

**DOI:** 10.3390/ncrna2040014

**Published:** 2016-12-04

**Authors:** Nazanin Karimnia, Haloom Rafehi, Natasha K. Tuano, Mark Ziemann, Harikrishnan K.N., Jun Okabe, Assam El-Osta

**Affiliations:** 1 Epigenetics in Human Health and Disease Laboratory, Baker IDI Heart and Diabetes Institute, The Alfred Medical Research and Education Precinct, Melbourne, Victoria 3004, Australia; Nazanin.Karimnia@bakeridi.edu.au (N.K.); Haloom.rafehi@bakeridi.edu.au (H.R.); Natasha.tuano@bakeridi.edu.au (N.K.T.); Mark.Ziemann@bakeridi.edu.au (M.Z.); Harikrishnan.Kaipananickal@bakeridi.edu.au (H.K.N.); jun.okabe@bakeridi.edu.au (J.O.); 2 Department of Pathology, The University of Melbourne, Parkville, Victoria 3010, Australia; 3 Faculty of Medicine, Nursing and Health Sciences, Monash University, Victoria 3004, Australia; 4 Central Clinical School, Monash University, Melbourne, Victoria 3004, Australia; 5 Hong Kong Institute of Diabetes and Obesity, Prince of Wales Hospital, The Chinese University of Hong Kong, Hong Kong SAR, China

**Keywords:** Set7, stem cell differentiation, non-coding RNA, histone, non-histone protein

## Abstract

Set7 is a key regulatory enzyme involved in the methylation of lysine residues of histone and non-histone proteins. This lysine methyltransferase is induced during stem cell differentiation and regulates lineage specific gene transcription and cell fate. In this article we discuss recent experimental evidence identifying regulatory targets under the control of Set7 as well as emerging evidence of regulation in stem cell differentiation. Furthermore, we discuss the function of non-coding RNAs regulated by Set7 implicated in cell plasticity.

## 1. Introduction

Histone lysine methylation has emerged as a key epigenetic signature in the transcriptional regulation of gene expression [1]. As such, lysine methyltransferase enzymes catalyse the methyl-writing ability at specific lysine residues of histone and non-histone proteins and have important regulatory roles in defining both gene activation and repression. Set7 was originally identified as mono-methyltransferase with writing ability on lysine 4 of histone H3 (H3K4me1) and was predominantly associated with open chromatin formation and thus permissive in gene expression [2,3]. Set7 also could efficiently control transcriptional events independent of histone modification to directly methylate transcription factors that are recruited at promoters [4]. In fact, many non-histone targets have been identified that not only belong to a family of transcriptional factors but also include chromatin modifiers and RNA binding proteins [5,6]. The challenge now is to understand the diverse substrate specificity of Set7 with that of biological function and one notable example of this increasing complexity is hyperglycemic memory. Set7 serves as a sensor of hyperglycemia controlling persistent activation of pro-inflammatory genes in the vasculature [7,8]. The inner working of gene regulation under glucose stimulation is remarkably complex with Set7 playing a critical role in defining the expression of genes implicated in inflammation. Indeed, glucose stimulation of vascular endothelial cells directionally controls histone and non-histone mediated mechanisms of regulation by the Set7 enzyme [8]. This broad substrate specificity is as diverse as the regulatory proteins and the biological actions that are under control. The effectiveness of extracellular stimuli to regulate signalling pathways relies on the capacity of Set7 to modify target molecules. This cellular response is clearly observed during stem cell differentiation as well as early development of the zygote. Typically, genes involved in the maintenance of pluripotency are downregulated, whereas those genes that are responsible for cell fate are often upregulated. This regulatory switch is controlled by chromatin organization and the specific binding of transcription factors that serve to control gene expression. Moving into lineage specificity and cell fate control, several groups have recently shown that Set7 expression is increased during stem cell differentiation [9,10,11,12], suggesting that its activity is a critical switch in cell differentiation. As there have been several outstanding articles that discuss Set7 function in metabolism [13], transcriptional control [6] and genome stability [5,6], we chose to focus our attention on the recent experimental results that implicate Set7-mediated stem cell differentiation. In this perspective we discuss some of the conflicting phenotypes that have been described using Set7 knockout mice studies with the aim to understand the broad functional role of the enzyme. Finally, we explore the possible role for Set7 in the regulation of transcription factors and non-coding RNAs (ncRNAs) implicated in embryonic stem cell (ESC) differentiation.

## 2. Non-Histone Methylation by Set7 and Genetic Set7 Knockout Mouse Studies

The structural specificity of SET domain methyltransferase studies has dramatically expanded with non-histone substrates for the Set7 methyltransferase. Based on the catalytic domain and crystal structure, the substrate and product specificities of the Set7 enzyme reveal remarkable plasticity in binding [4,14,15]. Peptide array experiments assessing methylation show Set7 recognises the sequence motif: [G/R/H/K/P/S/T]–[K>R]–[S>K/Y/A/R/T/P/N]–K–[Q/N]–[A/Q/G/M/S/P/T/Y/V] in peptide (target lysine for methylation is underlined) [14]. Because of the broad substrate specificity, Set7 has a widespread regulatory role in transcriptional signalling [4]. For instance, the stability and degradation of p53 is regulated in part by Set7-mediated lysine methylation and plays a critical role in the response to DNA damage [15,16]. While this study went on to show Set7 mediated methylation of p53 protein, it is also reported that half of the homozygous Set7 knockout (Set7KO) mice had died during embryogenesis [16] (Table 1).

Independent studies investigating p53-dependant transcriptional regulation show the homozygous knockout of the Set7 allele in mice are viable without causing marked consequences on tumor suppressor activity [17,18]. While reasonable explanations to resolve these discordant findings remain elusive, these studies indicate an important developmental role for Set7 in embryogenesis that is distinct from its activity in adult tissue. More recent studies using Set7KO mice have found important roles in the regulation of the pathways such as Hippo/Yes-Associated Protein (YAP) [19], and TGF-**β** signalling [20]. Further studies are required to characterize the role of the lysine methyltransferase during development and in the signalling pathways linked with embryogenesis.

## 3. Set7 Regulates Stem Cell Differentiation

The transition of stem cells to a more differentiated state depends heavily on the precise programm of gene expression mediated by the interaction of transcription factors and epigenetic regulators. Recent studies have shown that Set7 expression is strongly upregulated during differentiation in myoblast cells [9], embryoid bodies [10], human and mouse ESCs [11,12]. Pluripotent transcription factors, Oct4 and Sox2 are critical in maintaining ESCs and reprogramming somatic cells into induced pluripotent stem cells [21]. Set7 methylates Sox2 protein to inhibit transcriptional activity and induces Sox2 degradation [10]. Indeed, Set7 expression is subject to control by the binding of Oct4 and Sox2 at its promoter during mouse ESC (mESC) differentiation [12]. These results suggest that activation of Set7 by degradation of pluripotency factors is important for facilitating cell differentiation. Set7 is a dynamic protein that determines subcellular localisation of its target protein(s) by methylation. For example, Set7 is translocated into the nucleus in human endothelial cells stimulated by hyperglycemia [8] and promotes cytoplasmic retention by monomethylation of YAP in the Hippo pathway [19]. Monomethylation of pluripotent factor LIN28A by Set7 is specifically localized in the nucleoli, which prevents nuclear biogenesis of the primary transcript of let-7, thereby regulating differentiation of human ESCs (hESCs) [22]. Thus, Set7 regulates activity and subcellular localization of the target proteins in stem cell differentiation.

Knockdown of Set7 (Set7KD) results in differentiation defects in various types of stem cells. Set7KD hESCs delay differentiation with defects in both the silencing of pluripotent markers and the induction of differentiation-associated genes [11]. Set7KD myoblast impairs skeletal muscle myocyte differentiation by loss of interaction with transcriptional factor MyoD protein [9]. Set7KD Sca1+ cells impair smooth muscle (SM) cell differentiation with global downregulation of SM-associated genes regulated by H3K4me1 as well as interaction with serum response factor (SRF) protein [12]. Therefore Set7 coordinates the expression of downstream factors necessary for differentiation through methylation of histone and non-histone proteins.

## 4. Is Set7 Restricted to SM-Associated Gene Regulation?

**Figure 1:**
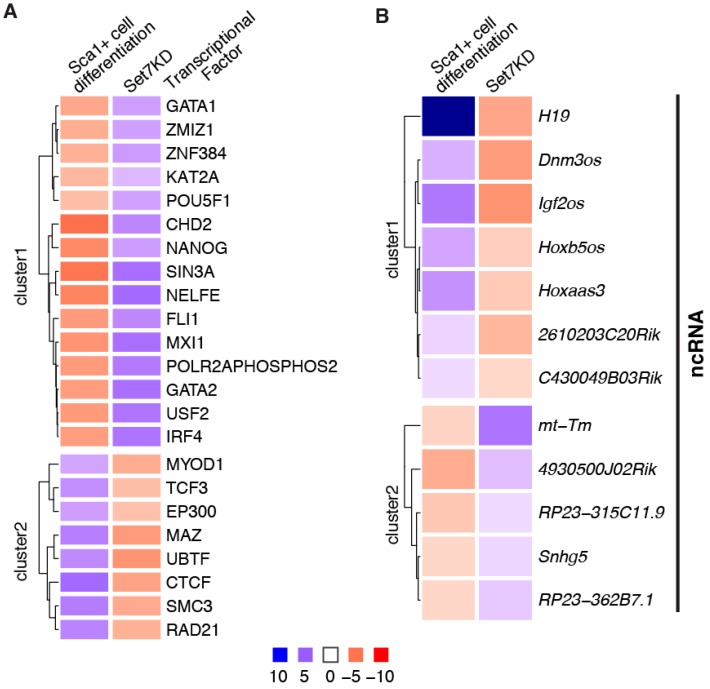
Set7 regulates activity of transcriptional factors and the expression of non-coding RNA in mESC differentiation. RNA-seq data from mESC, Sca1+ cells and Sca1+ Set7KD cells were accessed from GEO (GSE81830 [12]) and aligned to the mouse genome (mm10, ensembl release 77). Differential gene expression was determined using edgeR. Two comparisons were performed: the Sca1+ cell differentiation model in which Sca1+ cells were compared to mESCs, and the Set7KD model in which Sca1+ Set7KD cells are compared to Sca1+ cells transfected with non-target vector. (**A**) Transcription factor (TF) analysis was performed using Gene Set Enrichment Analysis (GSEA) combined with TF ChIP-seq gene sets from mouse cell types derived from the ENCODE project [23]. The results are expressed as the Normalised Enrichment Score (NES). A positive NES indicates that genes with binding sites for the TF in question are generally increased in expression, while a negative NES indicates suppression of genes with a TFBS. (**B**) The heatmap summarises gene expression profiles of non-coding RNAs differentially expressed during differentiation and by Set7KD in Sca1+ cells, with a False Discovery Rate (FDR) *p* value < 0.1.

Transcriptional network analysis has shown that Set7 regulates differentiation-associated genes in SM, heart, adipose, skeletal muscle and brain [12]. In addition, TF analysis has demonstrated that Set7 is not restricted to the regulation of tissue-specific TFs (Figure 1A). Knockdown of Set7 in embryoid bodies have increased expression of ectoderm markers and reduced expression of endoderm markers [10]. In hESCs, the pluripotent markers such as SOX2 and OCT4 were increased but the genes associated with liver (HNF4) and blood (SOX6) were decreased by Set7KD during differentiation [11]. These studies suggest that the gene targets of Set7 are dependent on specific extracellular stimuli to regulate signalling pathways in differentiation.

## 5. Set7 Regulates the Expression of Differentiation-Associated ncRNA

MicroRNAs (miRNAs) are integral regulatory elements in the transcriptional control of gene expression. Because Set7 controls the processing of let-7 miRNA by methylation of LIN28A in hESCs [22], one can postulate that Set7 may regulate other ncRNAs during stem cell differentiation. To identify ncRNA genes during mESC differentiation we re-analysed RNA-seq data in Set7KD cells [12]. The analysis showed 26 ncRNAs differentially expressed in Set7KD Sca1+ cells and many were strongly associated with mESC differentiation. Indeed, ncRNAs known to be induced during development include H19, Igf2os [24], Dnm3os [25], 2610203C20Rik [26] and Hoxaas3 [27] were downregulated in Set7KD Sca1+ cells (Figure 1B). While the results require further investigation for biological activity the data implicate Set7 in the regulation of ncRNAs during mESC differentiation. 

## 6. Conclusions and Future Prospects

**Figure 2:**
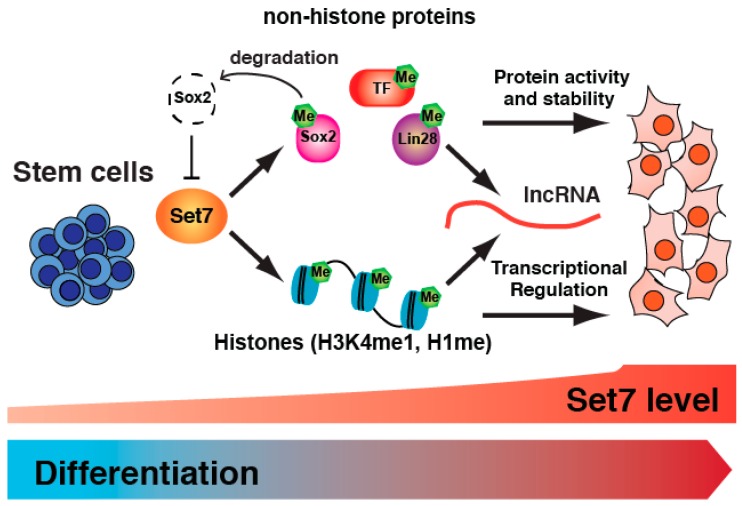
Set7 mediated stem cell differentiation. Recent studies reported that Set7 is upregulated during stem cell differentiation. Histones and non-histone proteins modified by Set7 exhibit changes in protein activity, stability and transcriptional regulation of genes during differentiation. For instance, methylated Sox2 protein by Set7 induces degradation of Sox2 protein followed by activation of the Set7 gene. Abundant Set7 promotes the broad expression of differentiation-associated genes. In addition, ncRNAs regulated by Set7 might be involved in differentiation and development.

Recent studies have unveiled the regulatory machineries of Set7 mediated stem cell differentiation (Figure 2).

The upregulation of Set7 might be a critical switch facilitating cell differentiation through transcriptional regulation, activity and degradation of Set7 target proteins. Furthermore, we propose that Set7 induces the expression of ncRNAs associated with development and differentiation. Little is known about the regulatory events, binding specificity and dynamics of many of the Set7 associated complexes that carry out ncRNA functions. While Set7 has been shown to bind RNA [28] and is implicated in its processing [22], the mechanisms regulating these events are yet to be fully elucidated. Strongly implicated but of unknown function in differentiation, the potential involvement of Set7 in the regulation of ncRNAs will require sophisticated molecular strategies to assess transcript recognition and enzymatic function during stem cell differentiation. This fascinating link also carries some important challenges to experimentally assess whether validated extracellular conditions such as hyperglycemia activates pro-inflammatory ncRNA targets. While metabolic memory has been associated with H3K4me1 and the persistent up-regulation of pro-inflammatory pathways in vascular cells [7], the way stem cells use this epigenetic information might determine cell fate because of the dramatic changes in Set7 activity. Recent studies have shown that AMI-5, a non-selective protein methyltransferase inhibitor enables Oct4-induced reprogramming of embryonic fibroblasts [29]. Since AMI-5 inhibits the activities of arginine and lysine methyltransferases the regulatory mechanisms involved are yet to be elucidated. Because AMI-5 inhibits enzyme activity in an AdoMet/SAM (S-adenosylmethionine) competitive manner [30], more selective inhibition of Set7 activity could efficiently reprogram somatic cells. In partnership with Pfizer, the Structural Genomics Consortium (SGC) developed PFI-2, a highly selective and cell-active inhibitor of Set7 [31]. PFI-2 occupies the lysine binding site of Set7 and interacts with the departing methyl group of SAM. Some of the connections between Set7 inhibition and stem cell differentiation are starting to be revealed at the molecular level. Studies have shown that PFI-2 delays Oct4 silencing and differentiation of hESCs [11]. More recently, we have shown this pharmacological inhibitor reduces the expression of SM-associated genes [12]. While these experiments suggest Set7 methyltransferase initiates or promotes cell determination, the challenge now will be to experimentally assess whether PFI-2 perturbs epigenetic memory during stem cell plasticity.

## Figures and Tables

**Table 1:** Comparison of the Set7 knockout mouse studies published.

Publication	Year	Knockout Type	Deletion Site	Knockout Strategy	Survival	Other Knockout Phenotype
Kurash et al. [16]	2008	Constitutive	Exon 2	Insertion of promoter less LacZ-Neo-poly-A cassette	Half of Set7 -/- mice died during embryogenesis.	Set7KO mice survived to adulthood appeared grossly normal. Set7KO could not induce p53 downstream targets upon DNA damage.
Lehnertz et al. [17]	2011	Conditional	Exon 4-8	Crossing to an actin-Flp deleter strain	Viable with no gross abnormality	Normal ability to p53 mediated cell cycle arrest and apoptosis following genotoxic stimuli in Set7KO mice
Campaner et al. [18]	2011	Constitutive	Exon 2	Red/ET-based recombineering	Viable with normal life span No increased predisposition to tumorigenesis	No effect on p53 dependent cell cycle arrest and apoptosis following DNA damage
Oudhoff et al. [19]	2013	Conditional	Exon 2	Intestinal epithelial cells (IECs) specific deletion	No overt phenotype	Shorter and wider intestinal crypts. Increase expression of YAP target genes in IECs
Elkouris et al. [20]	2016	Constitutive	Exon 4	Crossing to a CMV-Cre strain	Normal development and fertile	Set7KO has a protective effect against pulmonary fibrosis

## Abbreviations

The following abbreviations are used in this manuscript:

Set7: SET domain containing lysine methyltransferase 7
H3K4me1: Histone 3 lysine 4 monomethylation
TFs: Transcriptional Factors
ncRNA: non-coding RNA
ESC: Embryonic Stem Cell
KO: Knock Out
YAP: Yes-Associated Protein
TGFß: Transforming Growth Factor beta
Sox2: Sry box-containing gene 2
Oct4: Octamer-binding transcriptional factor 4
LIN28A: Lin-28 Homolog A
KD: Knock Down
RNA-Seq: RNA Sequencing
let-7: Lethal-7
MYOD: Myogenic differentiation protein
SM: Smooth Muscle
SRF: Serum Response Factor
HNF4: Hepatocyte Nuclear Factor 4
miRNA: microRNA
Sca1: Stem cell antigen 1
Igf2os: Insulin-like growth factor 2, opposite strand
Dnm3os: Dynamin 3, Opposite Strand
Hoxaas3: HOXA Cluster Antisense RNA 3
